# METTL3/YTHDF1‐Driven SURF6 Promotes Prostate Cancer Stemness via CDK4

**DOI:** 10.1111/jcmm.71259

**Published:** 2026-06-23

**Authors:** Yue Cheng, Min Zhang, Danfang Shi, XuFen Xia

**Affiliations:** ^1^ Department of Clinical Laboratory Tongde Hospital of Zhejiang Province Hangzhou China

**Keywords:** CDK4, m^6^A modification, PCa, proliferation, SURF6

## Abstract

Prostate cancer (PCa) remains a major health challenge globally, necessitating the identification of novel therapeutic targets to improve patient outcomes. This study investigates the role of the SURF6 gene in PCa, focusing on its expression patterns, molecular mechanisms, and biological behaviours in vitro and in vivo. We employed a combination of bioinformatics analysis, gene expression profiling, Western blotting, and functional assays, including cell proliferation, migration, invasion, and stemness assays, to evaluate the impact of SURF6 modulation in PCa cell lines. Our findings demonstrate that SURF6 is significantly upregulated in PCa tissues compared to adjacent normal tissues, with elevated expression correlating with poor prognosis and advanced clinical stages. Functional assays revealed that silencing SURF6 inhibited PCa cell proliferation, migration, and invasion, while overexpression of SURF6 enhanced these malignant characteristics. Notably, we identified that SURF6 regulates CDK4 expression, which plays a pivotal role in cell cycle progression and maintenance of stem‐like properties, as evidenced by the modulation of cancer stem cell markers such as CD44 and Nanog. Furthermore, we elucidated that METTL3 and YTHDF1 regulate SURF6 expression through m^6^A modification. In vivo experiments confirmed that knockdown of SURF6 inhibits tumour growth and reduces stemness features in xenograft models. Overall, our study underscores the critical role of SURF6 in promoting PCa progression and highlights its potential as a therapeutic target, paving the way for future research focused on targeting SURF6 in clinical settings to improve treatment strategies for PCa.

## 1 Introduction

Prostate cancer is one of the most prevalent malignancies affecting men worldwide, and its rising incidence poses significant public health challenges. The complexity of prostate cancer biology, particularly in relation to tumour progression and metastasis, necessitates a deeper understanding of the molecular mechanisms underlying these processes [1, 2]. Recent studies have identified a range of genetic and epigenetic alterations associated with prostate cancer, yet the specific contributions of various genes to tumour biology remain inadequately explored [3]. Among these, the SURF6 gene has emerged as a potential player in the pathogenesis of prostate cancer, given its established roles in ribosome biogenesis and cell proliferation across various cancer types [4].

Although SURF6 has been implicated in the progression of other malignancies, its specific role in prostate cancer has not been thoroughly investigated. SURF6 is a highly conserved nucleolar matrix protein that binds nucleic acids, participates in early pre‐rRNA processing, and contributes to ribosome biogenesis and cell‐cycle control [4, 5, 6, 7]. Dysregulation of nucleolar proteins and ribosome production is a hallmark of rapidly proliferating tumour cells and has been linked to aberrant G1–S transition and oncogenic transformation in several cancer types. Given these established nucleolar functions, SURF6 represents a biologically plausible candidate linking nucleolar activity to the aggressive behaviour of prostate cancer cells [7, 8]. Epitranscriptomic regulation by N6‐methyladenosine (m^6^A) has emerged as a pervasive layer of post‐transcriptional control that reshapes oncogenic gene‐expression programs [9]. m^6^A marks are installed by the METTL3‐METTL14 writer complex, in which METTL3 serves as the catalytic subunit, and METTL14 contributes to complex integrity and RNA substrate binding [10]. These m^6^A‐modified transcripts are then decoded by reader proteins; notably, YTHDF1 recognises m^6^A sites and promotes translation efficiency by engaging the translation machinery. In cancer contexts, dysregulated METTL3/YTHDF1 signalling has been associated with enhanced translation of pro‐tumorigenic targets and accelerated tumour progression [11].

The present study was designed to test the hypothesis that SURF6 is aberrantly upregulated in prostate cancer and that it promotes tumour progression and cancer stem cell–like properties by sustaining CDK4‐dependent cell‐cycle progression. In parallel, we posited that SURF6 expression is reinforced post‐transcriptionally through N6‐methyladenosine (m^6^A) RNA modification, whereby METTL3 deposits m^6^A marks on SURF6 mRNA that are recognised by the reader protein YTHDF1 to enhance SURF6 mRNA stability or translation. To address this central question, we combined pan‐cancer and prostate cancer bioinformatics analyses with in vitro gain‐ and loss‐of‐function experiments and in vivo xenograft models to define the expression pattern and clinical relevance of SURF6 in prostate cancer, determine its impact on proliferation, migration, invasion, and stemness, and dissect the METTL3/YTHDF1–SURF6–CDK4 axis that mechanistically connects nucleolar function to prostate cancer biology.

Furthermore, the study will explore the regulatory mechanisms governing SURF6 expression, particularly focusing on the role of N6‐methyladenosine (m^6^A) RNA modification, which has emerged as a significant post‐transcriptional regulatory mechanism in cancer biology [12]. By understanding how SURF6 expression is modulated through m^6^A modification pathways, we can identify novel therapeutic strategies that target these regulatory networks to inhibit prostate cancer progression.

In summary, this research seeks to fill the existing knowledge gap by investigating the functional role of SURF6 in prostate cancer. Our findings could provide valuable insights into the molecular mechanisms of prostate cancer progression and the potential of SURF6 as a therapeutic target, contributing to the ongoing efforts to develop more effective treatment strategies for this prevalent disease.

## 2 Result

### 2.1 Elevated SURF6 Expression as a Potential Therapeutic Target in Prostate Cancer

**FIGURE 1:**
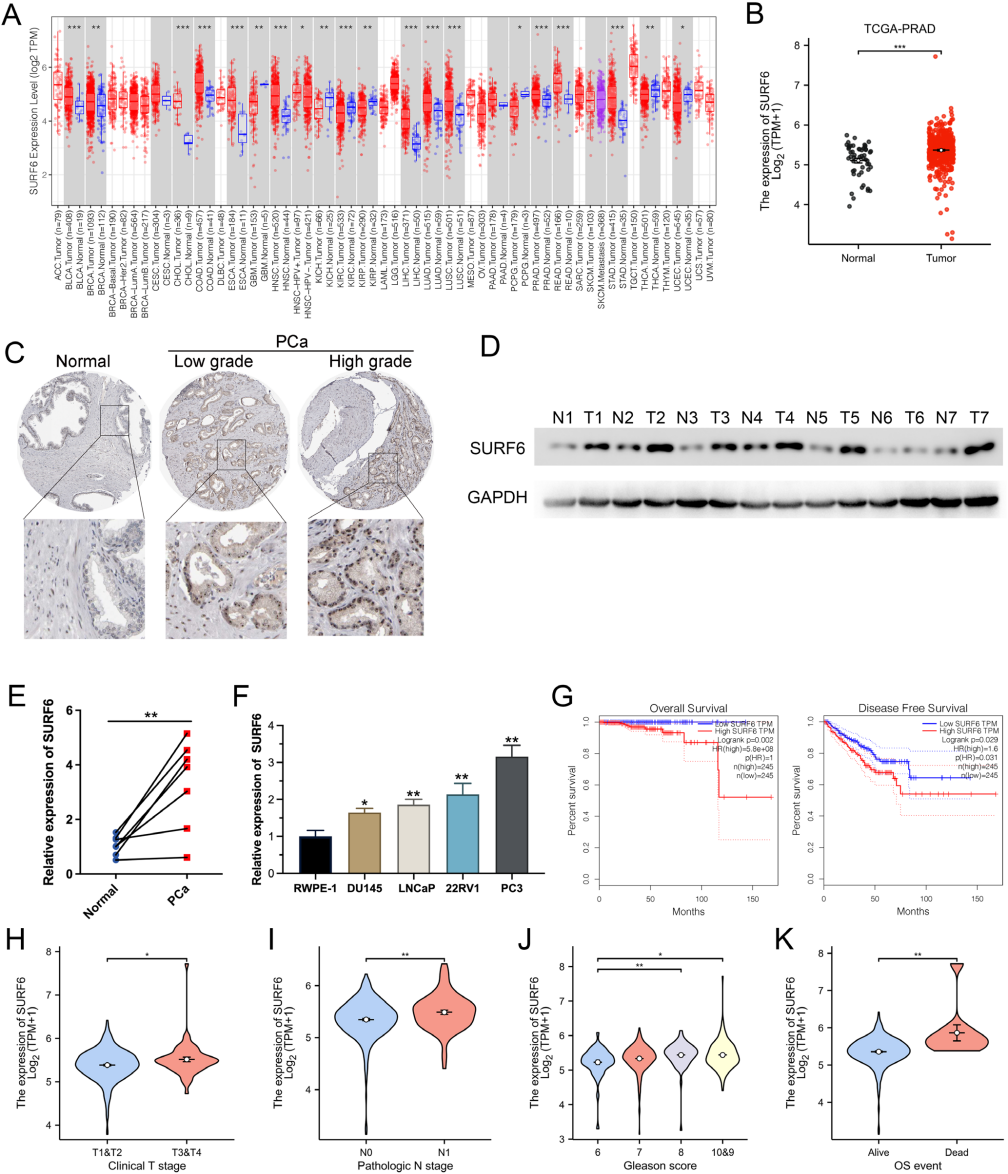
Elevated SURF6 expression as a potential therapeutic target in prostate cancer. (A) Analysis of SURF6 mRNA expression across various cancer types using the TIMER2.0 database. (B) Validation of high SURF6 expression in prostate cancer samples using the TCGA database. (C) Immunohistochemical (IHC) staining from the Human Protein Atlas shows increased SURF6 levels in prostate cancer tissues compared to normal tissues, with higher expression in high‐grade tumours. (D, E) Western blotting and RT‐qPCR demonstrate elevated SURF6 protein and mRNA levels in tumour tissues vs. adjacent normal tissues. (F) RT‐qPCR analysis indicates significantly higher SURF6 mRNA levels in prostate cancer cell lines compared to RWPE‐1 normal prostate epithelial cells. (G) Overall Survival (OS) and Disease‐Free Survival (DFS) analysis using GEPIA2 demonstrates that high expression of SURF6 is significantly associated with poor prognosis in prostate cancer (PCa) patients; (H) Analysis of TCGA clinical data showing that SURF6 expression is markedly higher in T3 & T4 stage PCa compared to T1 & T2 stage; (I) Elevated SURF6 expression in N1 stage PCa relative to N0 stage; (J) Patients with a Gleason score of ≥ 8 exhibit a higher risk score than those with a Gleason score of = 6; (K) OS event analysis indicates that high SURF6 expression correlates with increased mortality among PCa patients.

To investigate the differential expression of SURF6 between tumours and adjacent normal tissues, we analysed the mRNA expression levels of SURF6 across various cancer types using the TIMER2.0 database. The results indicated that SURF6 is significantly upregulated in bladder urothelial carcinoma (BLCA), invasive breast cancer (BRCA), cholangiocarcinoma (CHOL), colon adenocarcinoma (COAD), oesophageal cancer (ESCA), head and neck squamous cell carcinoma (HNSC), hepatocellular carcinoma (LIHC), prostate adenocarcinoma (PRAD), rectal adenocarcinoma (READ), and stomach adenocarcinoma (STAD) in comparison to adjacent normal tissues. Conversely, reduced expression of SURF6 was observed in glioblastoma multiforme (GBM), clear cell renal carcinoma (KIRC), kidney papillary cell carcinoma (KIRP), pheochromocytoma and paraganglioma (PCPG), thyroid carcinoma (THCA), and uterine corpus endometrial carcinoma (UCEC) (Figure 1A). Based on our extensive research on prostate cancer, we further validated the high expression of SURF6 in prostate cancer tissues using the TCGA database (Figure 1B). To further examine the protein expression of SURF6, we retrieved representative immunohistochemical (IHC) staining data from the Human Protein Atlas (HPA). The results demonstrated a significant upregulation of SURF6 in PCa samples compared to normal tissues, with higher expression levels in high‐grade tumours compared to low‐grade tumours (Figure 1C). Additionally, we analysed the protein and mRNA levels of SURF6 in frozen tissue samples of tumours and paired adjacent normal tissues using Western blotting and RT‐qPCR. The findings revealed that SURF6 expression was higher in tumour tissues than in adjacent normal tissues (Figure 1D,E). We also examined the expression of SURF6 in PCa and normal cell lines. RT‐qPCR analysis of SURF6 mRNA levels in GC cell lines and RWPE‐1 prostate epithelial cells showed significantly elevated SURF6 mRNA levels in PCa cell lines compared to RWPE‐1 cells (Figure 1F). These results suggest that SURF6 may serve as an important therapeutic target for prostate cancer.

### 2.2 Prognostic Value and Subgroup Analysis of SURF6 in Prostate Cancer

Furthermore, analysis of Overall Survival (OS) and Disease‐Free Survival (DFS) of prostate cancer (PCa) patients using GEPIA2 revealed that high expression of SURF6 is closely associated with poor prognosis (Figure 1G). We also downloaded clinical data and gene expression profiles for PCa from TCGA to investigate the relationship between SURF6 expression and clinical‐pathological parameters in PCa patients. The results indicated that SURF6 expression was significantly higher in T3 & T4 stage PCa compared to T1 & T2 stage (Figure 1H). Additionally, SURF6 expression was markedly elevated in N1 stage PCa compared to N0 stage (Figure 1I). Patients with a Gleason score of ≥ 8 had a higher risk score compared to those with a Gleason score of = 6 (Figure 1J). Moreover, in the OS event analysis of PCa patients, high expression of SURF6 was also found to be associated with patient mortality (Figure 1K).

### 2.3 SURF6 Regulates Malignant Biological Behaviours of PCa Cells in Vitro

**FIGURE 2:**
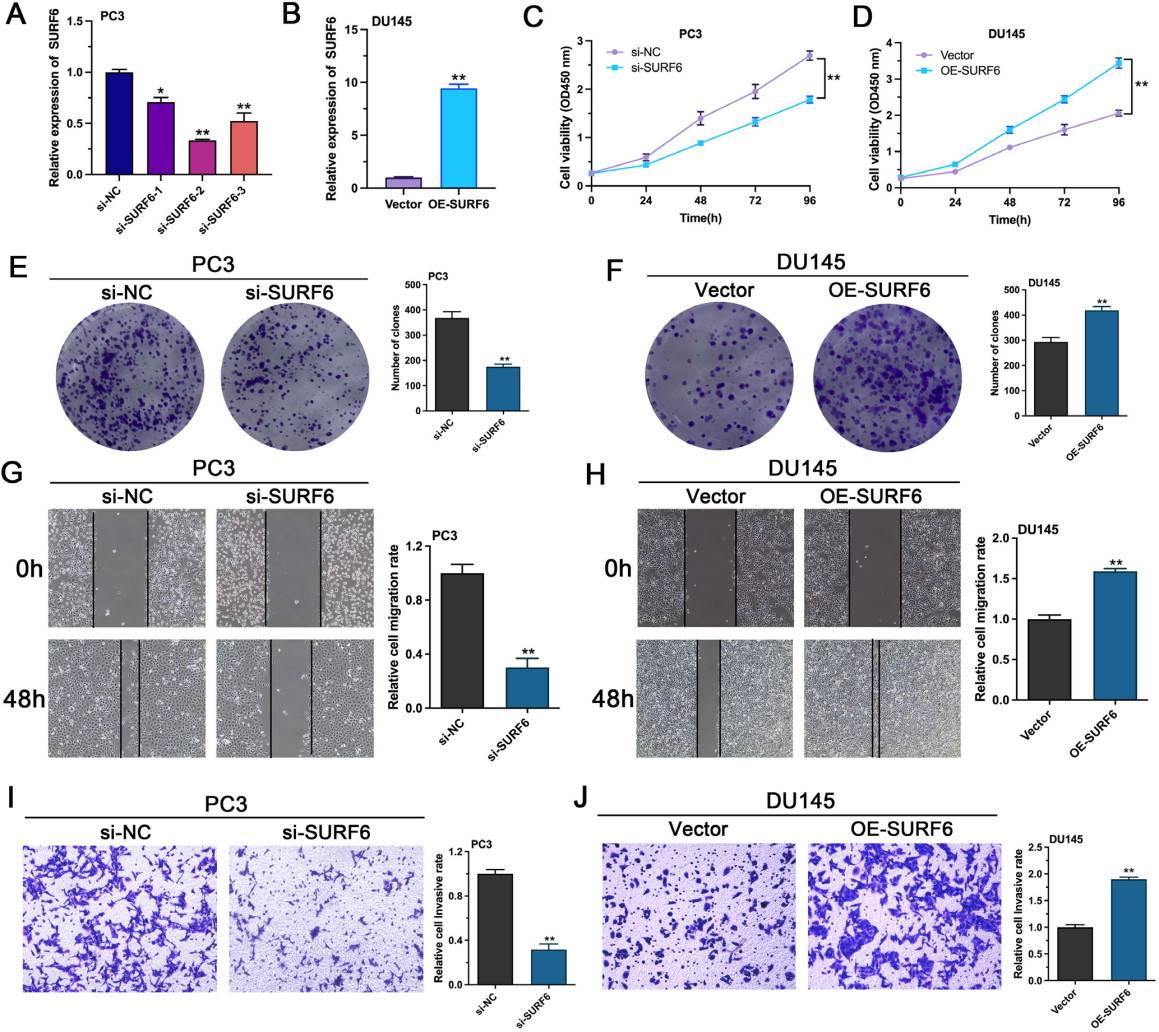
SURF6 regulates malignant biological behaviours of PCa cells in vitro. (A, B) RT‐qPCR analysis confirming the efficiency of SURF6 knockdown in PC3 cells using siRNA and the successful overexpression of SURF6 in DU145 cells via an overexpression plasmid; (C) CCK‐8 assay demonstrating that knocking down SURF6 significantly inhibits the proliferation of PC3 cells compared to the si‐NC group; (D) CCK‐8 assay showing that overexpression of SURF6 markedly enhances the proliferation of DU145 cells relative to the Vector group; (E, F) Colony formation assays illustrating the role of SURF6 in promoting PCa cell proliferation; (G) Wound healing assay indicating that silencing SURF6 significantly suppresses the migration of PC3 cells compared to the si‐NC group; (H) Wound healing assay revealing that overexpression of SURF6 enhances the migratory capacity of DU145 cells compared to the Vector group; (I) Transwell assay demonstrating that silencing SURF6 notably inhibits the invasion ability of PC3 cells relative to the si‐NC group; (J) Transwell assay showing that overexpression of SURF6 facilitates the migratory capacity of DU145 cells compared to the Vector group. Data are presented as means ± SD, *n* = 3, ***p < 0.001; **p < 0.01; *p < 0.05.

To investigate the role of SURF6 in prostate cancer (PCa), we utilised siRNA to knock down SURF6 expression in PC3 cells, while also constructing a SURF6‐overexpressing DU145 cell line using an overexpression plasmid. The interference efficiency of siRNA and the effect of the overexpression plasmid were analysed by RT‐qPCR (Figure 2A,B). CCK‐8 assays demonstrated that knocking down SURF6 expression significantly inhibited the proliferation of PC3 cells compared to the si‐NC group (Figure 2C). In contrast, overexpression of SURF6 markedly promoted the proliferation of DU145 cells when compared to the Vector group (Figure 2D). Colony formation assays further revealed the role of SURF6 in promoting PCa cell proliferation (Figure 2E,F). Subsequently, wound healing assays indicated that silencing SURF6 expression significantly suppressed the migration of PC3 cells compared to the si‐NC group (Figure 2G). Conversely, overexpression of SURF6 significantly enhanced the migratory capacity of DU145 cells compared to the Vector group (Figure 2H). Similarly, in Transwell assays, silencing SURF6 expression notably inhibited the invasion ability of PC3 cells relative to the si‐NC group (Figure 2I). Overexpression of SURF6 also significantly facilitated the migration capacity of DU145 cells compared to the Vector group (Figure 2J). Collectively, these results suggest that higher SURF6 expression promotes the progression of PCa.

### 2.4 Role of SURF6 in Regulating Cancer Stem Cell‐Like Characteristics of PCa Cells

**FIGURE 3:**
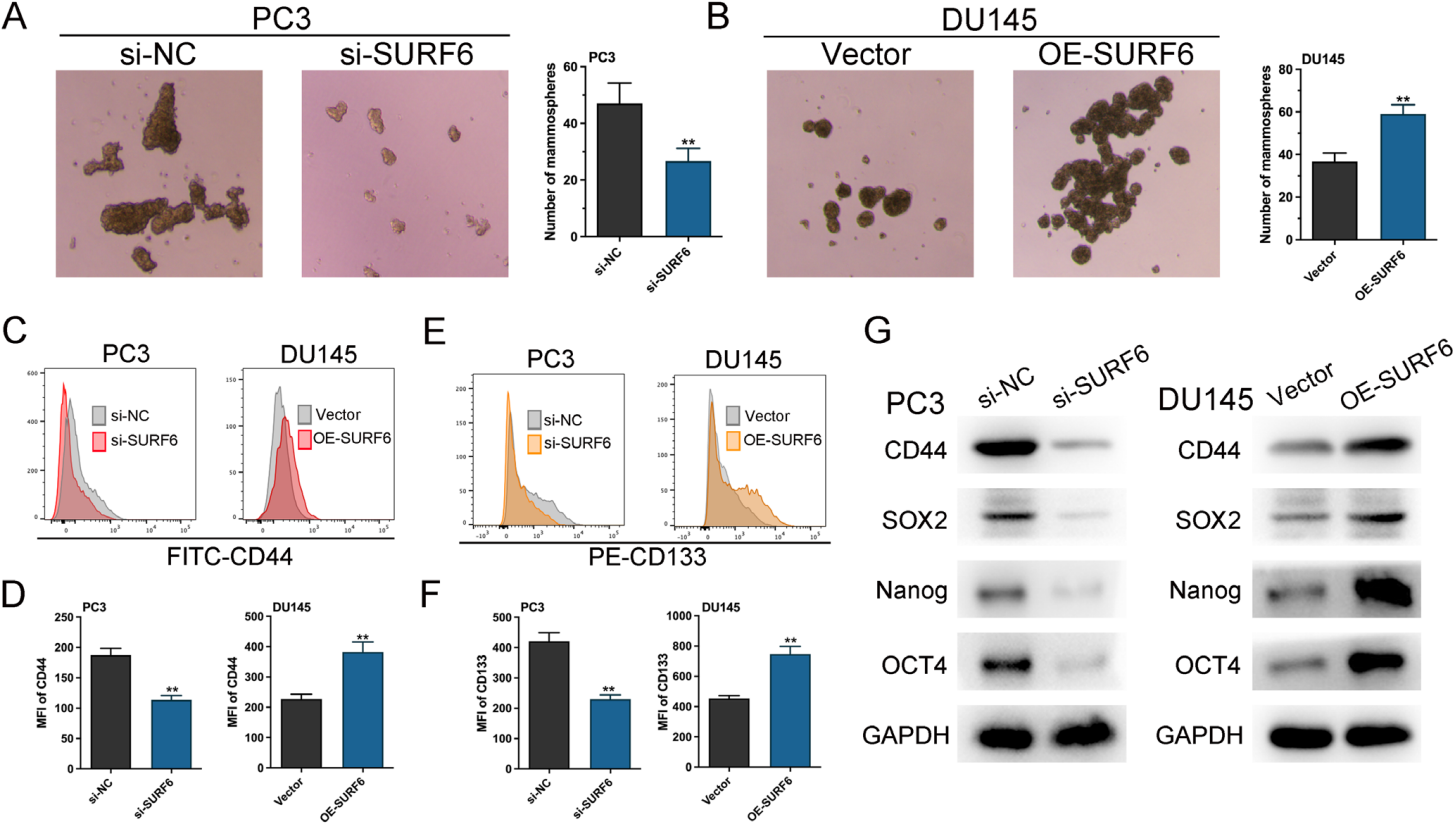
Role of SURF6 in regulating cancer stem cell‐like characteristics of PCa Cells. (A) Sphere formation assay demonstrating that knocking down SURF6 significantly inhibits the sphere‐forming ability of PC3 cells compared to the si‐NC group; (B) Sphere formation assay showing that overexpression of SURF6 markedly enhances the sphere‐forming capability of DU145 cells relative to the Vector group; (C–F) Flow cytometry analysis revealing a significant reduction in CD44^+^ and CD133^+^ populations in SURF6‐knockdown PC3 cells, while an increase in these populations is observed in SURF6‐overexpressing DU145 cells; (G) Western blot analysis of stemness markers including CD44, SOX4, Nanog, and OCT4, indicating that silencing SURF6 results in decreased expression of these markers, whereas overexpression of SURF6 leads to their increased expression. Data are presented as means ± SD, *n* = 3, ***p < 0.001; **p < 0.01; *p < 0.05.

Cancer stem cells (CSCs) are a distinct population of cells characterised by their self‐renewal and tumorigenic potential. To elucidate the role of SURF6 in the stem‐like characteristics of PCa cells, we performed sphere formation assays, which are a hallmark of CSCs [13, 14]. Results showed that knocking down SURF6 expression significantly inhibited the sphere‐forming ability of PC3 cells compared to the si‐NC group (Figure 3A). Conversely, overexpression of SURF6 markedly enhanced the sphere‐forming capability of DU145 cells when compared to the Vector group (Figure 3B). Research has reported that CD44 and CD133 serve as cell surface markers for prostate cancer stem cells (PCSCs). Consistent with these findings, a significant reduction in the populations of CD44^+^ and CD133^+^ cells was observed in SURF6‐knockdown PC3 cells, whereas a notable increase in these populations was detected in SURF6‐overexpressing DU145 cells (Figure 3C–F). Additionally, Western blot analysis of stemness markers such as CD44, SOX4, Nanog, and OCT4 after modulating SURF6 levels showed that knocking down SURF6 led to a reduction in these stemness markers, whereas overexpressing SURF6 resulted in their increased expression, indicating changes in stem cell characteristics (Figure 3G). Collectively, these results suggest that SURF6 plays a crucial role in regulating the stem cell‐like features of PCa cells in vitro.

### 2.5 METTL3 And YTHDF1 Regulate SURF6 Expression via m^6^A Modification

**FIGURE 4:**
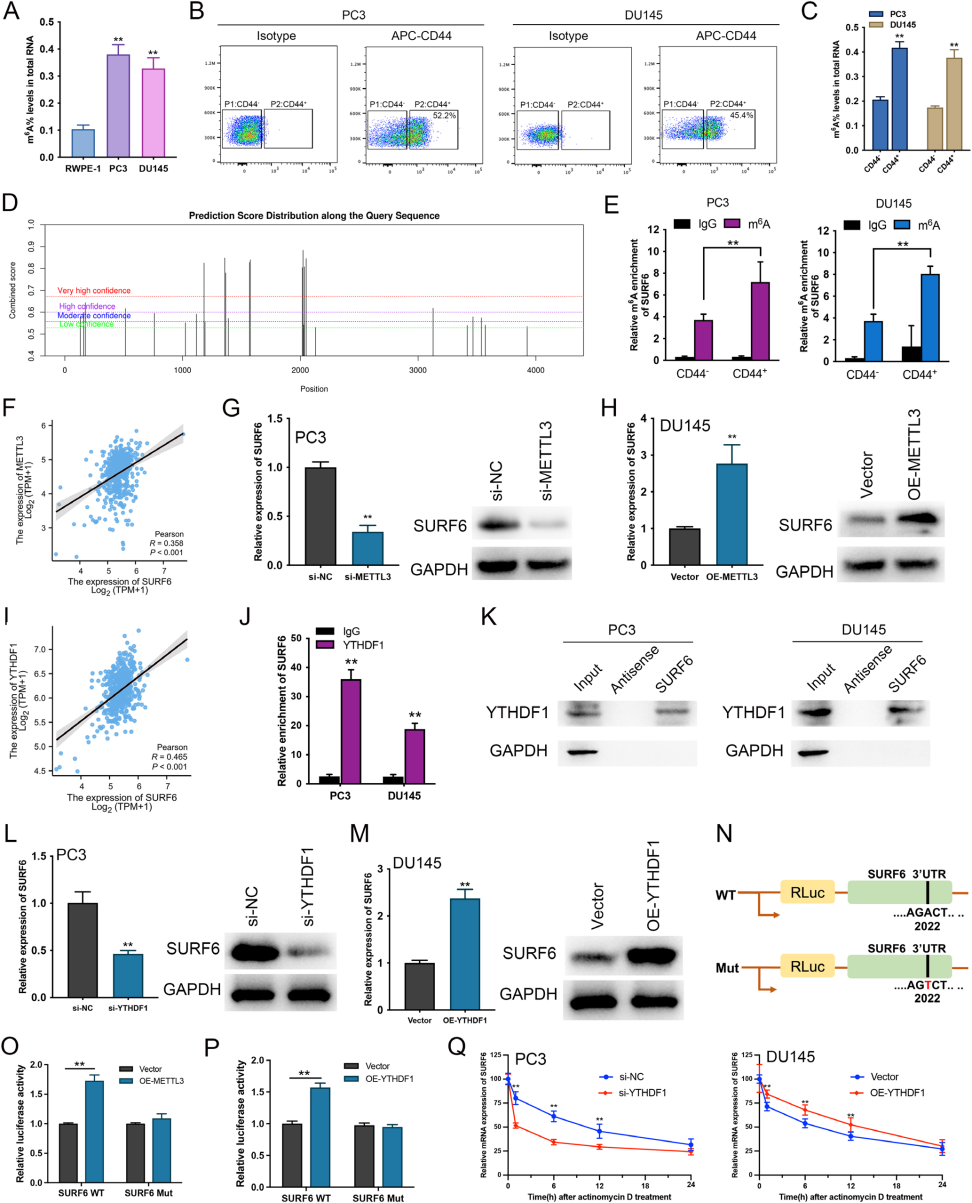
METTL3 and YTHDF1 regulate SURF6 expression via m^6^A modification. (A) Comparison of overall m^6^A levels in normal prostate epithelial cells (RWPE‐1) vs. prostate cancer cells (PC3 and DU145), showing significantly higher m^6^A levels in cancer cells; (B) FACS sorting of CD44^−^ (P1) and CD44^+^ (P2) cells in PC3 and DU145 using APC–anti‐CD44, with isotype controls setting the negative gate; (C) Analysis of m^6^A levels in CD44^+^ and CD44^−^ populations within PC3 and DU145 cells, indicating markedly elevated m^6^A in the CD44^+^ population; (D) Identification of high‐confidence m^6^A modification sites within the SURF6 mRNA sequence using SRAMP; (E) RNA immunoprecipitation (m^6^A‐RIP) results demonstrating significant enrichment of SURF6 in the CD44^+^ cell population compared to the control IgG group; (F) TCGA analysis revealing a significant correlation between SURF6 and METTL3 expression; (G, H) RT‐qPCR and Western blot analyses confirming that siRNA‐mediated knockdown of METTL3 in PC3 cells leads to decreased SURF6 expression, while overexpression of METTL3 promotes SURF6 levels in DU145 cells; (I) TCGA analysis indicating a significant correlation between SURF6 and YTHDF1 expression; (J) RNA immunoprecipitation (RIP) confirming the interaction between YTHDF1 and SURF6 mRNA, with significant enrichment observed in both PC3 and DU145 cells compared to the control IgG group; (K) RNA pull‐down showing YTHDF1 binding to SURF6 RNA in PC3 and DU145; antisense served as a negative control; (L) Knockdown of YTHDF1 in PC3 cells leading to downregulation of SURF6 at both RNA and protein levels; (M) Overexpression of YTHDF1 enhancing SURF6 expression; (N) Schematic of WT and m^6^A‐site mutant (Mut) SURF6 3′UTR luciferase reporters; (O, P) Dual‐luciferase assay: METTL3 (O) or YTHDF1 (P) increased WT SURF6 3′UTR activity but not Mut; (Q) Stability analysis of SURF6 RNA post‐transfection with YTHDF1 siRNA and negative control siRNA, showing enhanced degradation of SURF6 RNA in the absence of YTHDF1, while its overexpression improves SURF6 RNA stability in DU145 cells. Data are presented as means ± SD, *n* = 3, ***p < 0.001; **p < 0.01; *p < 0.05.

Recent studies have increasingly demonstrated that m^6^A modification plays a critical role in the malignant biological behaviours of tumours and the maintenance of tumour cell stemness [15, 16]. Initially, we compared the overall m^6^A levels in normal prostate epithelial cells and PCa cells, revealing that the total RNA m^6^A levels in PC3 and DU145 were significantly higher than those in RWPE‐1 (Figure 4A). PC3 and DU145 cells were stained with APC‐anti‐CD44, with an APC‐isotype control setting the negative threshold, and then gated into CD44^−^ (P1) and CD44^+^ (P2) populations for FACS collection and downstream analyses (Figure 4B). Further comparisons between CD44^−^ and CD44^+^ populations in PC3 and DU145 cells indicated that the m^6^A levels were markedly elevated in CD44^+^ positive cells compared to the CD44^−^ cell population (Figure 4C). m^6^A modification is one of the most abundant RNA modifications involved in post‐transcriptional regulation of multiple processes. To explore whether SURF6 undergoes m^6^A modification, we analysed the m^6^A modification sites within the SURF6 mRNA sequence using SRAMP (http://www.cuilab.cn/sramp), identifying several high‐confidence m^6^A modification sites (Figure 4D). To further validate the m^6^A modification of SURF6, we performed RNA immunoprecipitation (m^6^A‐RIP) using an m^6^A antibody. The results showed that SURF6 was significantly enriched compared to the control IgG group, with higher enrichment observed in the CD44^+^ positive cell population than in the CD44^−^ group (Figure 4E). m^6^A is a reversible RNA modification that can be methylated by m^6^A methyltransferases and demethylated by m^6^A demethylases. Methyltransferases METTL14 and METTL3, as well as demethylases ALKBH5 and FTO, have been reported to regulate the m^6^A levels of RNA transcripts. Based on this, we conducted TCGA analysis and found a significant correlation between SURF6 and the expression of the methyltransferase METTL3 (Figure 4F). Additionally, siRNA‐mediated knockdown of METTL3 in PC3 cells resulted in a significant decrease in SURF6 expression, as evidenced by RT‐qPCR and Western blot analyses. Conversely, overexpression of METTL3 promoted SURF6 expression levels in DU145 cells (Figure 4G,H). Therefore, SURF6 undergoes m^6^A modification, which is regulated by METTL3. Research indicates that when m^6^A in current mRNAs is recognised by YTHDF, it can promote mRNA decay or translation, depending on the specific YTHDF involved. Through TCGA analysis, we discovered a significant correlation between SURF6 and YTHDF1 expression (Figure 4I). RNA immunoprecipitation (RIP) using a YTHDF1 antibody further confirmed the interaction between YTHDF1 and SURF mRNA, with SURF6 RNA being significantly enriched in both PC3 and DU145 compared to the control IgG group (Figure 4J). Consistently, an RNA pull‐down assay using SURF6 sense RNA retrieved YTHDF1 in both PC3 and DU145 cells, whereas the antisense control showed minimal binding (Figure 4K). To determine whether YTHDF1 affects SURF6 expression, we knocked down YTHDF1 in PC3 cells and observed downregulation of SURF6 at both RNA and protein levels (Figure 4L). Conversely, overexpression of YTHDF1 enhanced SURF6 expression (Figure 4M).

To validate the functional m^6^A site within the SURF6 3′UTR, we generated luciferase reporters containing the wild‐type (WT) SURF6 3′UTR or a mutant (Mut) construct with the putative m^6^A motif disrupted (Figure 4N). Dual‐luciferase assays showed that METTL3 overexpression significantly increased the activity of the WT SURF6 3′UTR reporter, whereas this effect was largely abolished in the Mut reporter (Figure 4O). Consistently, YTHDF1 overexpression enhanced luciferase activity driven by the WT SURF6 3′UTR but not the Mut construct (Figure 4P), indicating that this m^6^A site is required for METTL3/YTHDF1‐dependent regulation of SURF6. Moreover, we analysed the impact of YTHDF1 on the stability of SURF6 RNA. Following transfection of YTHDF1 siRNA and negative control siRNA into PC3 cells, total RNA was harvested at different time points after treatment with actinomycin D. The absence of YTHDF1 significantly enhanced the degradation of SURF6 RNA (Figure 4Q). In contrast, the overexpression of YTHDF1 in DU145 cells significantly improved the stability of SURF6 RNA. In summary, METTL3 and YTHDF1 regulate SURF6 expression through m^6^A modification.

### 2.6 SURF6 Regulates Biological Behaviour of Prostate Cancer by Modulating the Cell Cycle

**FIGURE 5:**
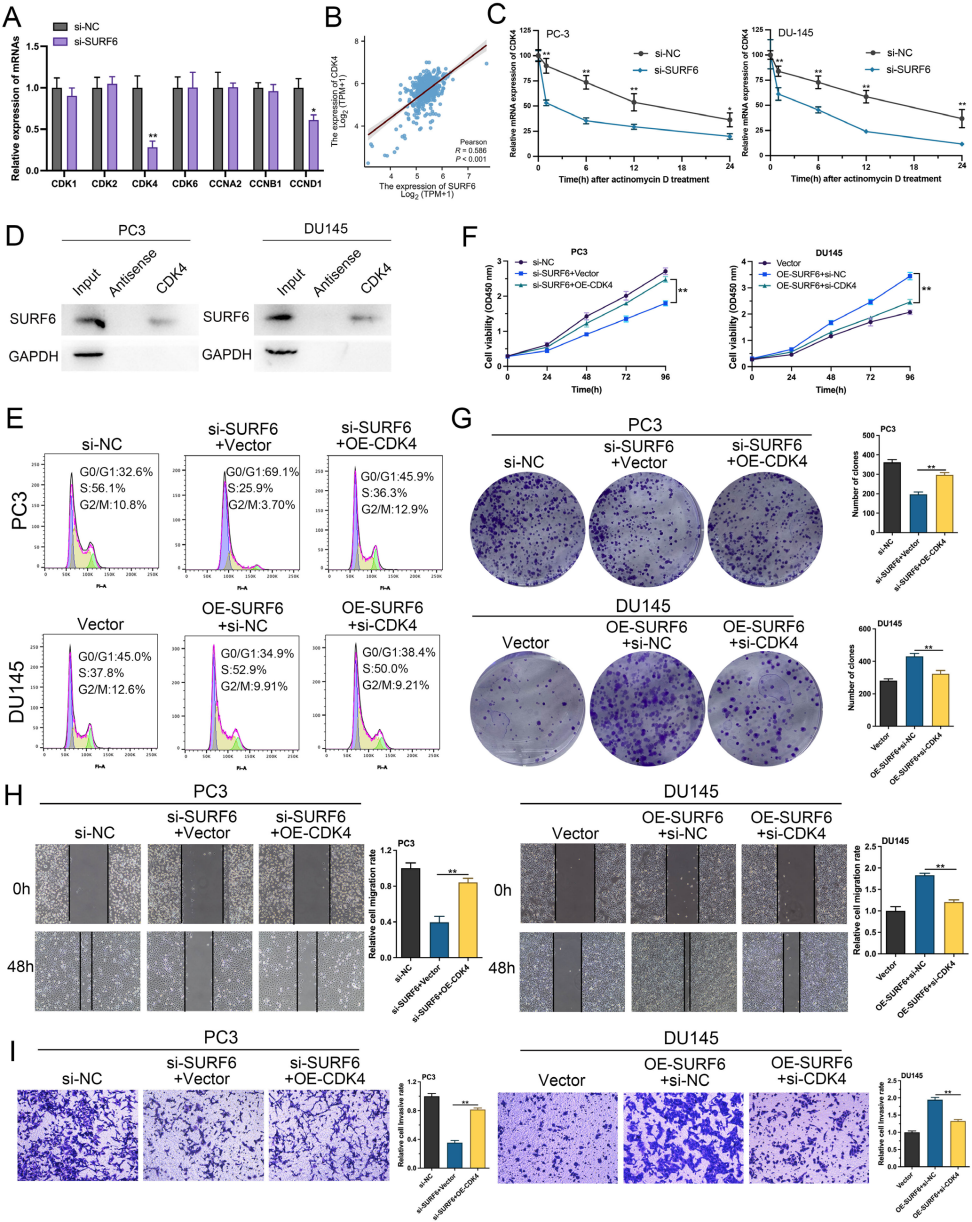
SURF6 regulates prostate cancer cell behaviour through CDK4 modulation and cell cycle dynamics. (A) Expression levels of CDK4 significantly decreased following SURF6 knockdown in prostate cancer cells. (B) Analysis of TCGA data indicates a notable correlation between SURF6 and CDK4 expression in prostate cancer samples. (C) Actinomycin D chase assay showing accelerated decay of CDK4 mRNA in SURF6‐silenced PC3 and DU145 cells compared with si‐NC controls. (D) RNA pull‐down assay demonstrating enrichment of SURF6 in complexes captured by CDK4 sense RNA in PC3 and DU145 cells; antisense RNA and input served as controls. (E) Flow cytometry results show that overexpression of CDK4 in PC3 cells mitigates the increase in G0/G1 phase cells due to SURF6 suppression, while CDK4 knockdown in DU145 cells reduces the decrease in G0/G1 phase cells induced by SURF6. (F, G) CCK8 and colony formation assays reveal that CDK4 overexpression rescues proliferation inhibition from SURF6 suppression in PC3 cells, whereas CDK4 knockdown in DU145 cells diminishes proliferation induced by SURF6. (H, I) Wound healing and Transwell assays demonstrate that CDK4 overexpression restores migratory and invasive capabilities suppressed by SURF6, while CDK4 knockdown in DU145 cells compromises migration and invasion prompted by SURF6. Data are presented as means ± SD, *n* = 3, ***p < 0.001; **p < 0.01; *p < 0.05.

Research has indicated that SURF6 plays a regulatory role in the cell cycle [7, 8]. To clarify whether SURF6 influences the biological behaviour and stemness maintenance of prostate cancer (PCa) by modulating the cell cycle, we examined the expression of cell cycle‐related molecules following SURF6 knockdown. Our results demonstrated a significant downregulation of CDK4 expression upon SURF6 knockdown (Figure 5A). Furthermore, TCGA data analysis revealed a significant correlation between SURF6 and CDK4 expression in prostate cancer (Figure 5B). To further determine whether SURF6 regulates CDK4 at the post‐transcriptional level, we assessed CDK4 mRNA decay following actinomycin D treatment. CDK4 mRNA declined more rapidly in SURF6‐silenced PC3 and DU145 cells than in control cells, indicating a reduced mRNA half‐life upon SURF6 knockdown (Figure 5C). Consistently, RNA pull‐down assays using CDK4 RNA enriched SURF6 in both PC3 and DU145 cells, whereas the antisense control showed minimal binding (Figure 5D), supporting a physical association between SURF6 and CDK4 transcripts.

To investigate the effect of SURF6 on the cell cycle, flow cytometry analysis was performed. Rescue experiments indicated that overexpression of CDK4 in PC3 cells mitigated the increase in G0/G1 phase cell proportion induced by SURF6 suppression. Conversely, knockdown of CDK4 in DU145 cells significantly reduced the decrease in G0/G1 phase cell proportion prompted by SURF6 (suggesting that SURF6 regulates the cell cycle by arresting cells in the G0/G1 phase) (Figure 5E). Moreover, CCK8 assays and colony formation rescue experiments demonstrated that CDK4 overexpression rescued the proliferation inhibition caused by SURF6 suppression in PC3 cells, while CDK4 knockdown in DU145 cells significantly attenuated the cell proliferation induced by SURF6 (Figure 5F,G). Similarly, wound healing and Transwell assays showed that CDK4 overexpression in PC3 cells rescued the migratory and invasive capabilities suppressed by SURF6, whereas CDK4 knockdown in DU145 cells markedly weakened the migratory and invasive effects induced by SURF6 (Figure 5H,I). Overall, our study demonstrates that SURF6 influences the biological behaviour of prostate cancer through its regulation of the cell cycle, primarily via modulation of CDK4 expression.

### 2.7 The Role of SURF6 in Maintaining Stemness of Prostate Cancer Through CDK4

**FIGURE 6:**
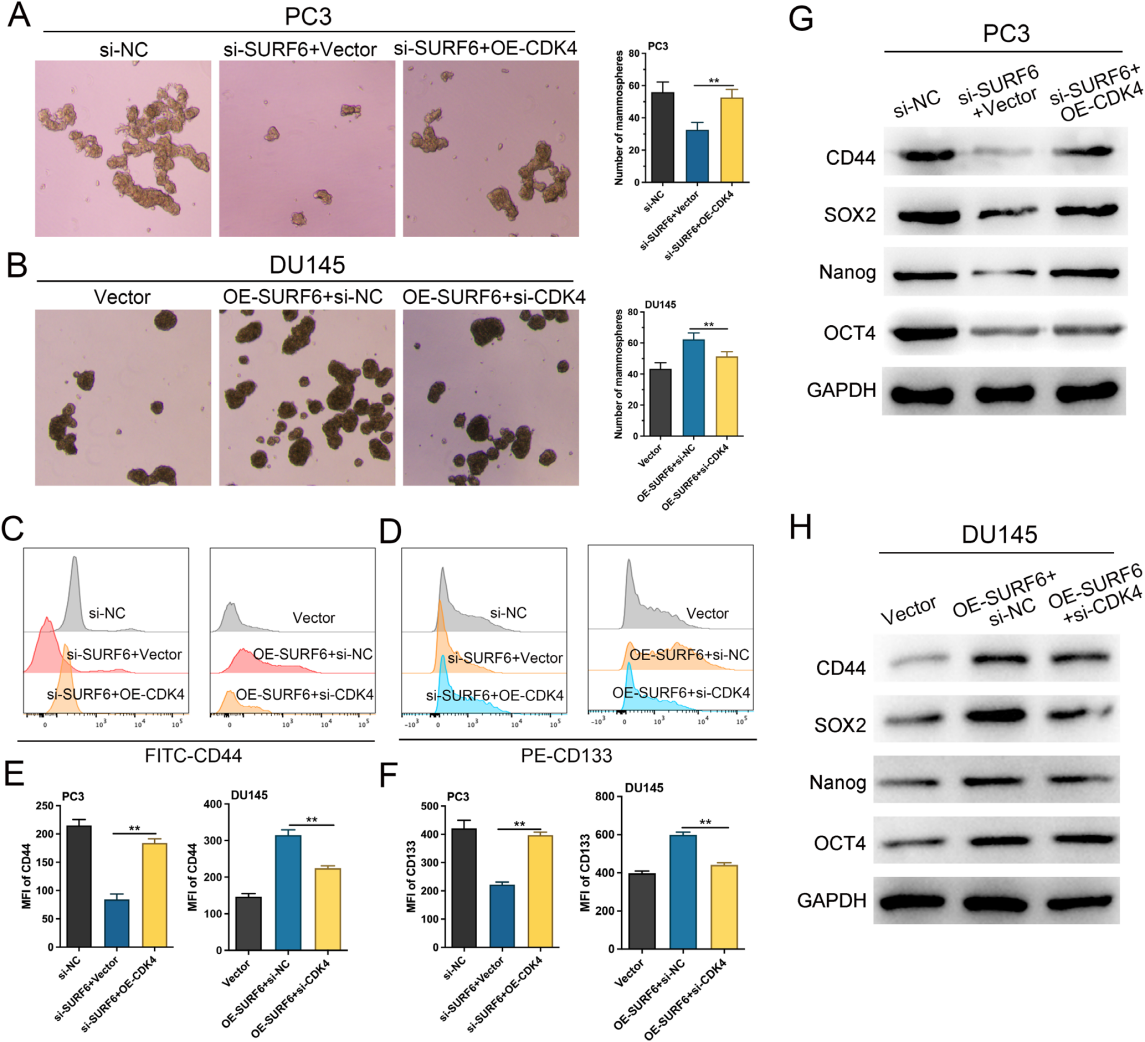
SURF6 maintains stemness in prostate cancer via CDK4 regulation. (A, B) Sphere formation assays show that overexpression of CDK4 rescues the impaired sphere‐forming ability in PC3 cells due to SURF6 suppression, while CDK4 knockdown in DU145 cells significantly reduces sphere‐forming capacity induced by SURF6. (C–F) Flow cytometry analysis indicates that CDK4 overexpression in PC3 cells restores the population of CD44^+^ and CD133^+^ cells diminished by SURF6 inhibition, whereas silencing CDK4 in DU145 cells attenuates the increase of these stemness markers promoted by SURF6. (G, H) Western blot analysis confirms that CDK4 overexpression in PC3 cells counteracts the downregulation of CD44, SOX4, Nanog, and OCT4 caused by SURF6 depletion, whereas CDK4 knockdown in DU145 cells significantly impairs the upregulation of these markers induced by SURF6. Data are presented as means ± SD, *n* = 3, ***p < 0.001; **p < 0.01; *p < 0.05.

Furthermore, we investigated the role of SURF6 in maintaining the stemness of prostate cancer (PCa) through CDK4. Sphere formation assays indicated that CDK4 overexpression rescued the impairment of sphere‐forming ability caused by SURF6 suppression in PC3 cells. Conversely, CDK4 knockdown in DU145 cells significantly reduced the sphere‐forming capacity induced by SURF6 (Figure 6A,B). Flow cytometry analysis revealed that CDK4 overexpression in PC3 cells rescued the reduction of CD44^+^ and CD133^+^ cell populations caused by SURF6 inhibition, while silencing CDK4 in DU145 cells diminished the increase of CD44^+^ and CD133^+^ cells induced by SURF6 (Figure 6C–F). Western blot analysis further confirmed that CDK4 overexpression in PC3 cells restored the downregulation of CD44, SOX4, Nanog, and OCT4 resulting from SURF6 depletion. In contrast, CDK4 knockdown in DU145 cells significantly weakened the upregulation of these markers elicited by SURF6 (Figure 6G,H). Overall, our results demonstrate that SURF6 regulates the stem‐like properties of PCa cells in vitro through the modulation of CDK4.

### 2.8 Knockdown of SURF6 Inhibits Prostate Cancer Growth in Vivo

**FIGURE 7:**
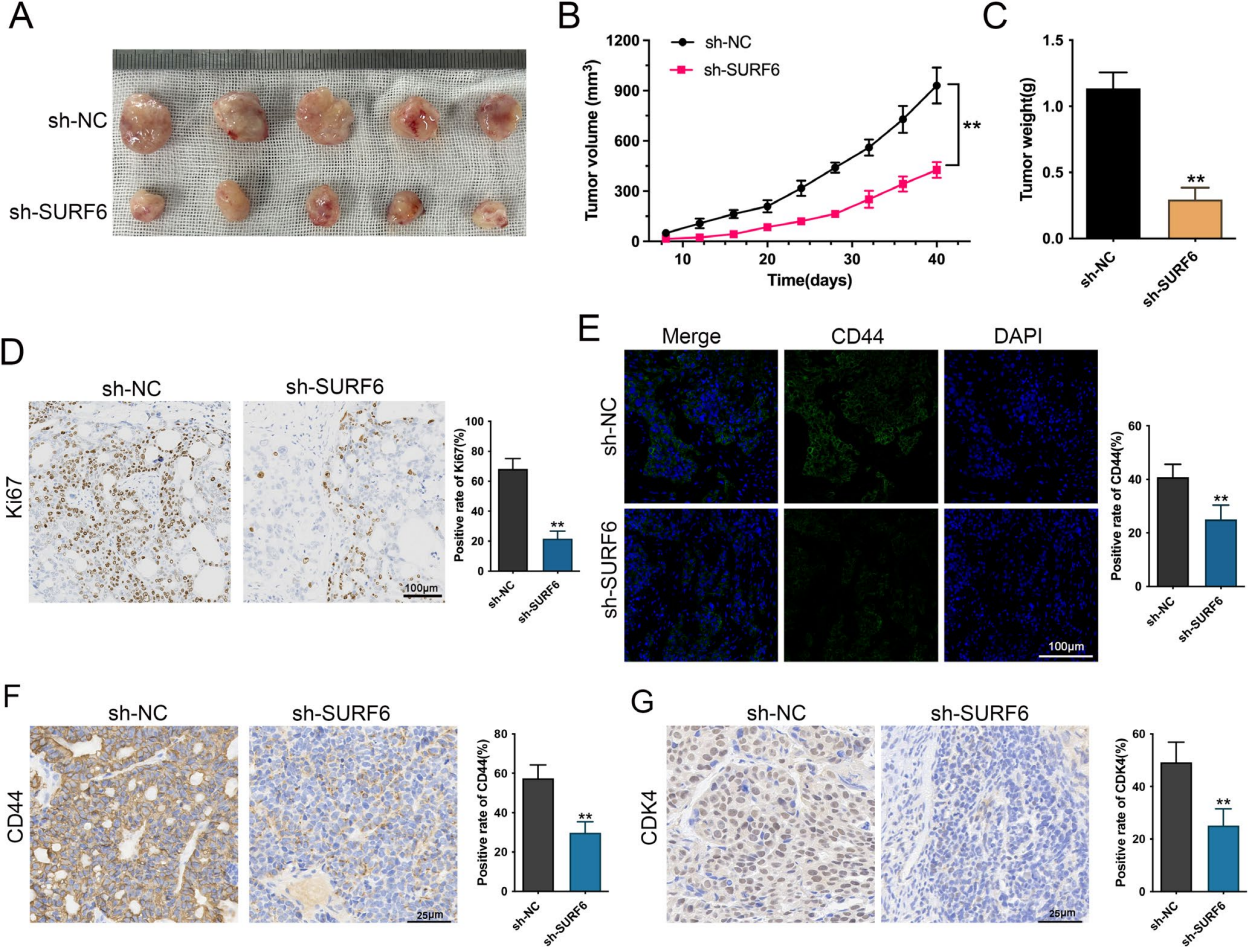
Knockdown of SURF6 inhibits prostate cancer growth in vivo. (A, B) Tumour volumes assessed every four days show that xenograft tumours derived from PC3 cells transfected with sh‐SURF6 are significantly smaller than those from the sh‐NC group during the study period. (C) Average tumour weight is markedly lower in the sh‐SURF6 group compared to sh‐NC. (D) Histological analysis reveals a significant decrease in Ki‐67 staining in sh‐SURF6 tumours, reflecting diminished proliferative activity relative to sh‐NC tumours. (E) Immunofluorescence staining shows a notable reduction in CD44 fluorescence intensity in the sh‐SURF6 group compared to the sh‐NC group. (F, G) IHC staining of CD44 and CDK4 in sh‐NC and sh‐SURF6 groups, with quantification of the positive rate. Data are presented as means ± SD, *n* = 3, ***p < 0.001; **p < 0.01; *p < 0.05.

To assess the impact of SURF6 on prostate cancer (PCa) proliferation in vivo, we implanted stable PC3 cells transfected with sh‐SURF6 and sh‐NC into nude mice. Tumour volumes were measured every four days post‐injection. Throughout the development of xenograft tumours, the tumour volume in the sh‐SURF6 group was significantly smaller than that in the sh‐NC group (Figure 7A,B). Additionally, the average tumour weight in the sh‐SURF6 group was markedly lower than in the sh‐NC group (Figure 7C). Histological analysis of tumour sections revealed a significant reduction in Ki‐67 staining, indicating decreased proliferative activity in the sh‐SURF6 tumours compared to the sh‐NC group (Figure 7D). Immunofluorescence staining further demonstrated a significant decrease in CD44 fluorescence intensity in the sh‐SURF6 group compared to the sh‐NC group (Figure 7E). Consistently, IHC analysis further confirmed that SURF6 knockdown markedly reduced CD44 and CDK4 staining compared with the sh‐NC group. Quantification of IHC signals showed significantly lower positive rates of CD44 and CDK4 in the sh‐SURF6 group (Figure 7F,G). These findings are consistent with our in vitro studies and strongly suggest that SURF6 contributes to the progression of PCa in vivo.

## 3 Discussion

Prostate cancer (PCa) is the most prevalent malignancy among men and continues to pose significant health challenges globally, being a leading cause of cancer‐related mortality. The complexity of its pathogenesis, combined with the heterogeneity of tumour behaviour, underscores the urgent need for a deeper understanding of the underlying molecular mechanisms [17, 18]. One area of particular interest is the role of cancer stem cells (CSCs), which are known to contribute to tumour initiation, progression, and recurrence due to their self‐renewal and differentiation capabilities [19, 20]. Recent studies have highlighted the importance of identifying specific markers and therapeutic targets associated with CSCs to develop more effective treatment strategies for PCa [21, 22].

This research investigates the elevated expression of the SURF6 gene in prostate cancer, aiming to delineate its role in tumour progression and its potential as a therapeutic target. By employing a combination of in vitro and in vivo methodologies, including gene knockdown and overexpression experiments, this study reveals the significant impact of SURF6 on the proliferation, migration, and stem‐like characteristics of prostate cancer cells. Furthermore, our findings suggest that SURF6 may regulate critical pathways associated with cell cycle progression, thereby influencing the aggressive nature of the tumour. The results highlight the potential of targeting SURF6 for therapeutic intervention in prostate cancer, opening avenues for future research aimed at improving patient outcomes [6].

The findings from this study reveal significant insights into the molecular mechanisms by which the SURF6 gene contributes to prostate cancer progression. Specifically, the upregulation of SURF6 in prostate cancer tissues correlates with poor prognosis and is associated with increased proliferation and migration of cancer cells. The elucidation of SURF6's role in regulating CDK4 expression, a key player in cell cycle progression, underscores the importance of this pathway in tumorigenesis [23]. This not only reinforces the hypothesis that SURF6 may act as an oncogene but also suggests that targeting the SURF6‐CDK4 axis could present a novel therapeutic strategy for managing prostate cancer, potentially offering a means to disrupt the aggressive characteristics associated with high SURF6 expression [24].

As the most abundant internal modification on eukaryotic mRNAs, N6‐methyladenosine (m^6^A) provides a dynamic layer of epitranscriptomic regulation that shapes RNA fate and gene expression programs [25]. METTL3, the catalytic core of the m^6^A “writer” complex, reshapes oncogenic gene‐expression programs by installing m^6^A marks on target transcripts [9]. These METTL3‐deposited m^6^A signals are decoded by YTHDF1, a canonical “reader” that typically enhances translation of methylated mRNAs, thereby amplifying pro‐tumorigenic signalling outputs [26]. Moreover, the investigation into the m^6^A modification of SURF6 provides a fresh perspective on its regulatory mechanisms. The study demonstrates that m^6^A methylation, influenced by METTL3 and YTHDF1, plays a crucial role in the expression of SURF6, which in turn impacts the malignant behaviours of prostate cancer cells [27, 28]. This highlights the potential for targeting m^6^A modification pathways as a therapeutic avenue, suggesting that modulation of these pathways could effectively regulate SURF6 levels and, consequently, the aggressive traits of prostate cancer cells. The interplay between m^6^A modification and cancer biology emphasises the need for further research into the broader implications of RNA modifications in cancer progression and treatment [12, 29].

The implications of SURF6 in regulating cancer stem cell‐like properties further extend the relevance of this research. The observed correlation between SURF6 expression and the maintenance of stemness markers such as CD44 and Nanog suggests that SURF6 is integral to the cancer stem cell population, which is critical for tumour initiation and recurrence [30, 31, 32]. Targeting SURF6 may offer a dual benefit by not only inhibiting tumour growth but also reducing the stem cell reservoir that contributes to treatment resistance. These findings pave the way for innovative treatment strategies that can effectively target the root of tumour recurrence, which is often associated with cancer stem cells.

In summary, this study identifies SURF6 as a pivotal regulator of prostate cancer progression, influencing critical biological behaviours such as cell proliferation, migration, and maintenance of stemness. The findings contribute to the understanding of the molecular underpinnings of prostate cancer and highlight the potential of targeting SURF6 for therapeutic intervention. By elucidating the role of SURF6 in regulating cancer stem cell characteristics and cell cycle dynamics, this research paves the way for future investigations aimed at developing novel treatment strategies that could improve patient outcomes in prostate cancer.

## 4 Materials and Methods

### 4.1 Collecting Clinical Samples and Culturing Cells

Tumour tissues and matched adjacent normal tissues were collected from 7 patients diagnosed with prostate cancer who underwent primary surgery at Tongde Hospital in Zhejiang Province between January 2022 and May 2024. All patients were diagnosed with prostate cancer based on pathological examination and had not received radiotherapy or chemotherapy prior to surgery. Clinical samples were collected immediately after resection and frozen using liquid nitrogen, then stored at −80°C. The study was approved by the Ethics Committee of Tongde Hospital in Zhejiang Province (No. 2026‐112 K), and written informed consent was obtained from all patients participating in the study. The prostate cancer (PCa) cell lines used in this study included DU145, LNCaP, 22RV1, PC3, and a human prostate cell line, RWPE‐1, all obtained from ATCC. All cells were cultured in RPMI 1640 medium containing 10% fetal bovine serum (derived from the blood of unborn calves) and maintained at 37°C in a humidified incubator with 5% CO_2_.

### 4.2 Bioinformatics Analysis

Pan‐cancer analysis of the SURF6 gene was performed using the TIMER2 database (http://timer.cistrome.org/). Expression data and clinical survival information related to SURF6 were extracted from the TCGA database (https://cancergenome.nih.gov/) and analysed using R software. The expression of SURF6 in prostate cancer tissues was analysed using the HPA database (https://www.proteinatlas.org/). m^6^A site prediction of RNA was performed using the SRAMP database (http://www.cuilab.cn/m6asiteapp/old).

### 4.3 Cell Transfection

si‐NC, si‐SURF6, si‐METTL3, si‐YTHDF1 small interfering RNAs and Vector, SURF6, METTL3, YTHDF1 overexpression plasmids were purchased from Shanghai Jima Biological. Si‐NC and Vector were used as control groups. Prior to transfection, PCa cell lines were plated in 6‐well plates for 24 h until the cell density reached 60%–70%. Then, cells were mixed with Lipofectamine 2000 (Invitrogen, USA) according to the manufacturer's protocol.

### 4.4 RT‐qPCR

We used the PrimeScript RT reagent kit (TAKARA, Japan) to convert mRNA into cDNA according to the manufacturer's protocol. The reaction conditions were as follows: 37°C for 15 min, then 85°C for 5 s. The cDNA products were immediately used as templates for PCR reactions. miRNA was reverse transcribed into cDNA using the Mir‐X miRNA First‐Strand Synthesis Kit (TAKARA, Japan) according to the manufacturer's protocol. The reaction conditions were as follows: 37°C for 60 min, then 85°C for 5 min. The cDNA products were immediately used as templates for PCR reactions. qPCR analysis was performed using the TB Green Premix Ex Taq kit according to the manufacturer's protocol. The reaction conditions were as follows: 95°C for 30 s, followed by 40 cycles of 95°C for 5 s and 60°C for 30 s. GAPDH was used as the internal reference. The relative expression levels were calculated using the 2^−ΔΔCT^ method. Primers were synthesised by Shanghai Sangon Biotech, with the following sequences:

SURF6‐F: CAGCCAAAGAGGAAGCAGCTTG.

SURF6‐R: CCTGGATCTTCTCATGCAGTCG.

METTL3‐F: CTATCTCCTGGCACTCGCAAGA.

METTL3‐R: GCTTGAACCGTGCAACCACATC.

YTHDF1‐F: CAAGCACACAACCTCCATCTTCG.

YTHDF1‐R: GTAAGAAACTGGTTCGCCCTCAT.

### 4.5 Western Blot

Cells were lysed on ice using RIPA lysis buffer (Beyotime, China) containing protease inhibitors. The lysate was centrifuged at 12,000 rpm for 10 min at 4°C, and the supernatant was collected. Protein concentration was determined using the BCA protein assay kit (Beyotime, China). Protein extract (30 μg) was separated by SDS‐PAGE and transferred to a PVDF membrane. The membrane was blocked with milk at room temperature for 2 h, then incubated overnight at 4°C with primary antibodies against GAPDH, SURF6, METTL3, YTHDF1, CD44, SOX2, Nanog, and OCT4 (CST, USA). The membrane was washed three times with TBST, then incubated with secondary antibodies (CST, USA) at 4°C for 2 h. The membrane was washed three more times with TBST and then developed using a chemiluminescence imaging system (Bio‐Rad, USA).

### 4.6 CCK‐8 Assay

According to the experimental grouping, PC3 and DU145 cells were transfected with siRNA or overexpression plasmids. After trypsinisation, cells were resuspended in fresh medium at 5 × 10^4^ cells/ml and seeded in 96‐well plates at 100 μL per well, with 6 replicates per group. Cells were cultured at 37°C and 5% CO2 for 24, 48, 72, and 96 h. Then, 10 μL of CCK‐8 reagent was added to each well, and incubation continued for 3 h. The absorbance of each well was measured at 450 nm using a microplate reader (Bio‐Rad, USA).

### 4.7 Colony Formation Assay

According to the experimental grouping, PC3 and DU145 cells were transfected with siRNA or overexpression plasmids, then seeded in 6‐well plates at a density of 1 × 10^4^ cells/ml. Cells were allowed to form colonies over a period of 14 days, with fresh medium replaced every 3 days. After cultivation, cells were fixed with 4% paraformaldehyde, stained with 0.5% crystal violet, and the number of colonies formed was counted under a microscope. The experiment was performed three times to ensure reproducibility.

### 4.8 Wound Healing Assay

According to the experimental grouping, PC3 and DU145 cells were transfected with siRNA or overexpression plasmids, then seeded in 6‐well plates at a concentration of 1 × 10^6^ cells/ml. Cells were allowed to adhere and grow overnight in serum‐free medium. A wound was created using a pipette tip, and the width of the wound was recorded. After 48 h of cell culture, the wound width was measured again to assess cell migration. Images were taken to record the changes in wound width over time. The experiment was performed three times to ensure reproducibility.

### 4.9 Transwell Assay

Matrigel was diluted to a final concentration of 5 μg/μl, and 50 μL/well was added to the upper chamber to form a gel. According to the experimental grouping, PC3 and DU145 cells were transfected with siRNA or overexpression plasmids, then trypsinised and resuspended in serum‐free medium at 1 × 10^6^ cells/ml. Then, 100 μL of the cell suspension was added to the upper chamber, and 700 μL of medium containing 10% FBS was added to the lower chamber. After 24 h of cell culture, the Transwell inserts were removed. Cells were washed twice with PBS and fixed with 4% paraformaldehyde for 10 min. Then, 0.1% crystal violet was added for staining for 10 min. After removing the crystal violet, cells were washed twice with PBS, and the upper surface cells were removed with a cotton swab. The cells attached to the lower surface were observed and photographed.

### 4.10 Flow Cytometry Analysis

Cell cycle detection was performed using Propidium Iodide (PI) staining. Cells from each experimental group in the logarithmic growth phase were collected, washed twice with PBS after trypsinisation, and then fixed in 70% pre‐cooled ethanol and stored in the dark at 4°C overnight. Fixed cells were washed twice with PBS and then stained with a solution containing RNase A (50 μg/mL) and PI (50 μg/mL) and incubated at 37°C for 30 min. Fluorescence intensity was detected using a flow cytometer (e.g., BD FACSCalibur) to identify the proportions of cells in different phases of the cell cycle (G0/G1, S, and G2/M), and data were analysed using FlowJo software. Detection of CD44 was performed using fluorescently labelled antibody staining. 1 × 10^6^ cells were collected, washed twice with PBS, and resuspended in 100 μL of flow cytometry buffer (PBS containing 1% BSA), followed by the addition of appropriate amounts of APC‐labelled anti‐CD44 antibody, and incubated in the dark at room temperature for 30 min. After incubation, cells were washed twice with PBS to remove unbound antibodies and resuspended in 500 μL of flow cytometry buffer, and data were collected using a flow cytometer and analysed using FlowJo software to determine the proportions of CD44^+^ cells. Unlabelled antibody cells were set as negative controls, and single‐labelled antibody groups were set for compensation settings.

### 4.11 RNA Pull‐Down

Biotin‐labelled SURF6 RNA probes (sense) and the corresponding antisense control were prepared by in vitro transcription and purified. PC3 and DU145 cells were lysed in ice‐cold lysis buffer supplemented with protease inhibitor cocktail and RNase inhibitor. Equal amounts of cleared lysates were pre‐cleared with streptavidin magnetic beads and then incubated with biotinylated RNA probes at 4°C with rotation. Streptavidin beads were subsequently added to capture RNA–protein complexes, followed by extensive washes with wash buffer to remove nonspecific binding. The bound proteins were eluted by boiling in SDS sample buffer and subjected to Western blotting using antibodies against YTHDF1; GAPDH was used as a negative/control marker where indicated.

### 4.12 Dual‐Luciferase Reporter Assay

The wild‐type (WT) SURF6 3′UTR fragment containing the putative m^6^A motif and the corresponding mutant (Mut) fragment were cloned downstream of the Renilla luciferase coding sequence in a dual‐luciferase reporter vector. PC3 and DU145 cells were seeded in 24‐well plates and co‐transfected with the WT or Mut reporter together with the indicated expression plasmids or corresponding control vectors (or siRNAs as specified) using a standard transfection reagent. At 24–48 h post‐transfection, luciferase activities were measured using a dual‐luciferase reporter assay kit according to the manufacturer's instructions. Renilla luciferase activity was normalised to Firefly luciferase activity as an internal control, and data were presented as relative luciferase activity compared with the corresponding control group.

### 4.13 Mammosphere Formation Assay

Cells were digested with trypsin to prepare a single‐cell suspension and filtered through a 40 μm cell strainer to obtain a uniform single‐cell suspension. Subsequently, single cells were seeded in ultra‐low attachment 6‐well plates at a density of 1 × 10^3^ cells/well. Cells were cultured under conditions containing DMEM/F12 medium (Gibco), 20 ng/mL epidermal growth factor (EGF), 20 ng/mL basic fibroblast growth factor (bFGF), 20 μL/mL B27 supplement (Gibco), and 4 μg/mL heparin for 10 days. The medium was changed every 3 days during the culture to avoid disturbing the cells. After cultivation, mammospheres were observed and photographed using an inverted microscope to record the formation and number of mammospheres. The experiment was repeated three times to ensure reproducibility of the results.

### 4.14 MeRIP‐qPCR and RIP‐qPCR

Approximately 20 million PC3 and DU145 cells treated in each experimental group were collected and washed with PBS. RNA immunoprecipitation (RIP) was performed using the Magna RIP Kit (Millipore) according to the manufacturer's protocol. Briefly, cells were lysed on ice in RIP lysis buffer containing protease inhibitors and RNase inhibitors for 60 min. Then, 50 μL of magnetic beads were coupled with anti‐m^6^A, anti‐YTHDF1, or anti‐rabbit IgG antibodies. The cell lysate was incubated overnight at 4°C with the magnetic bead‐antibody complex (precipitate). RNA immunoprecipitated from the precipitate was purified using phenol‐chloroform, and the immunoprecipitate was treated with proteinase K for RT‐qPCR.

### 4.15 RNA Stability Assay

Actinomycin D treatment was used to assess the stability of SURF6 mRNA. After 48 h of in vitro culture of transfected cells, actinomycin D was added at a final concentration of 5 μM to inhibit transcription. Cells were collected at 0 h, 1 h, 6 h, 12 h, and 24 h post‐treatment, and total RNA was extracted using TRIzol reagent. The extracted RNA was analysed by RT‐qPCR for SURF6 mRNA expression levels, using β‐actin mRNA as the internal reference gene. The stability of SURF6 mRNA was calculated by the ratio of its relative expression level to that of β‐actin mRNA, using the expression level at 0 h as a baseline to calculate the percentage of remaining mRNA at each time point. The experiment was repeated three times to ensure the accuracy and reproducibility of the results.

### 4.16 In Vivo Tumour Xenograft

Female BALB/c nude mice aged 4 weeks and weighing 20–24 g were purchased from Shanghai Slac Experimental Animal Co. Ltd. The nude mice were housed in a sterile SPF environment, with the temperature controlled at 24°C ± 2°C, and had free access to food and water. PC3 cells were transfected with SURF6 shRNA lentivirus. The transfected cells were harvested, resuspended in PBS, and subcutaneously injected (0.2 mL per mouse, *n* = 5 per group). Tumour length (a) and width (b) were measured using callipers, and tumour volume was calculated using the formula (a × b^2^)/2. After 4 weeks, the nude mice were euthanised, and tumours were collected, weighed, and stored at −80°C for further analysis. Tumour samples were also embedded in paraffin for histological analysis.

### 4.17 Immunohistochemistry (IHC)

Tumour tissues from nude mouse xenografts were fixed in 10% formaldehyde, embedded in paraffin, and cut into 4 μm sections. The sections were then dewaxed and dehydrated using xylene and a series of ethanol gradients. For immunohistochemistry (IHC) analysis, tumour tissue sections were treated with 3% H_2_O_2_ to inactivate endogenous enzymes, then heated to boiling in 0.01 M citrate buffer (pH = 6.0). After blocking, sections were incubated overnight at 4°C with Ki‐67, CD44 and CDK4 antibodies. The sections were then incubated with secondary antibodies, subjected to DAB staining, and counterstained with haematoxylin. Finally, the sections were dehydrated and mounted with neutral resin. Images were captured using an optical microscope.

### 4.18 Immunofluorescence (IF) Staining

Tumour tissues were fixed in 10% formaldehyde, embedded in paraffin, and sectioned at 4 μm. Sections were dewaxed and rehydrated, followed by antigen retrieval in citrate buffer (pH 6.0). After blocking with 5% BSA at room temperature, sections were incubated with anti‐CD44 primary antibody overnight at 4°C. After washing with PBS, sections were incubated with a fluorophore‐conjugated secondary antibody in the dark at room temperature. Nuclei were counterstained with DAPI, and images were captured using a fluorescence microscope.

### 4.19 Statistical Analysis

All data will be presented as mean ± standard deviation (SD). We will use a two‐tailed paired Student's *t*‐test to compare two groups of data and a one‐way ANOVA to compare at least three groups of data. Statistical analyses will be performed using GraphPad Prism 10 and SPSS 20.0, and statistical charts will be created using this software. To evaluate survival rates, we consider results significant if *p < 0.05, **p < 0.01, and ***p < 0.001. Each experiment will be conducted at least three times independently, and experimental data will be presented as mean ± standard deviation (SD).

## Author Contributions

**Yue Cheng:** writing – original draft, investigation, data curation. **Danfang Shi:** formal analysis, visualization. **Min Zhang:** methodology, formal analysis. **XuFen Xia:** writing – review and editing, project administration, resources, conceptualization.

## Funding

This work was supported by the Zhejiang Provincial Medical and Health Science and Technology Plan Project (No. 2024KY880), the Zhejiang Provincial Traditional Chinese Medicine Science and Technology Program (No. 2024ZR003), and the Basic Public Welfare Research Program of Zhejiang Province (No. LQ24H1290032).

## Ethics Statement

This study was approved by the Ethics Committee of Tongde Hospital in Zhejiang Province (No. 2026‐112 K).

## Consent

Informed consent obtained from all patients.

## Conflicts of Interest

The authors declare no conflicts of interest.

## Data Availability

The data that support the findings of this study are available from the corresponding author upon reasonable request.
